# Laryngeal granuloma occurring after surgery for laryngeal cancer treated by surgical removal and immediate post-operative radiotherapy: A case report

**DOI:** 10.1097/MD.0000000000017345

**Published:** 2019-09-27

**Authors:** Jingyi Wu, Tongchao Jiang, Yu Wu, Lijuan Ding, Lihua Dong

**Affiliations:** a Department of Radiotherapy; b Department of Otolaryngology, First Hospital of Jilin University, Changchun; c Department of Thoracic Oncology, Hubei Cancer Hospital, Wuhan, China.

**Keywords:** laryngeal granulomas, laryngeal neoplasm, radiotherapy, sugery

## Abstract

**Rational:**

Laryngeal granulomas are benign lesion that rarely occurs after surgery of laryngeal cancer. Until now there has not been standard treatment for it.

**Patient concern:**

The patient was diagnosed with laryngeal neoplasm one and half a month ago. Endoscopic low-temperature plasma knife in the radical excision of left vocal cord was performed under the general anesthesia. Postoperative histopathological examination confirmed left vocal cord tumor was highly differentiated invasive squamous cell carcinoma (SCC). Then the patient suffered unexplained intermittent dyspnea which persisted nearly 1 month after the surgery. Laryngoscope examination showed granulation formation on the glottis.

**Diagnoses:**

The patient was diagnosed with laryngeal granuloma 1 month after the surgery of laryngeal cancer.

**Interventions:**

The patient received resection of the laryngeal mass, and pathological examination confirmed the granuloma. Postoperative radiotherapy (RT) was performed within 24 hours after surgery.

**Outcomes:**

The patient was followed up for 3 years after surgery and the laryngeal granuloma and laryngeal cancer did not recur during follow-up. The symptoms of intermittent dyspnea disappeared and a satisfactory outcome was achieved.

**Lessons:**

Usually for primary laryngeal granulomas, surgical treatment alone is not enough, because it is easy to relapse. RT within 24 hours after operation can significantly reduce the recurrence of laryngeal granuloma.

## 1 Introduction

Laryngeal granulomas are uncommon benign disease with no exact cause yet.^[1]^ As we know, hyper functional vocal behaviors, intubation injury or reflux from the esophagus to the pharynx can contribute to the occurrence of the disease.^[2]^ However, laryngeal granulomas rarely occur after surgical resection of laryngeal cancer. To date, this is the first reported case of glottic granuloma occurring after laryngeal neoplasm operation.

Laryngeal cancer is the second most common type of head and neck malignancy worldwide, with estimated 151,000 new cases and 82,000 deaths annually in the world.^[3]^ SCC of the glottis is performed with surgery or RT depending on the extent of the disease.^[4]^

For primary granuloma, surgery remains as the standard treatment for laryngeal granuloma.^[5]^ However, surgical excision is associated with high recurrence rates.^[6,7]^ Low-dose postoperative RT has been proved capable of decreasing the recurrence rate of primary granuloma.^[1]^ As for laryngeal granuloma after laryngeal surgery, there is no standard treatment for this rare disease. We report this rare disease and use preventive dose radiotherapy (RT) within 24 hours after surgery to decrease the recurrence of laryngeal granuloma and laryngeal cancer.

## 2 Case report

**Figure 1:**
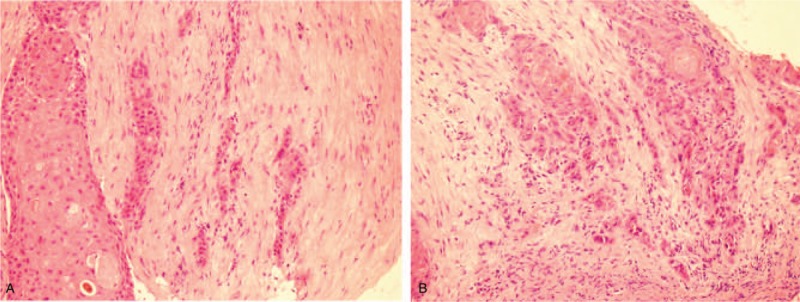
Hematoxylin and eosin staining. Microscopically, an invasive, highly differentiated squamous cell carcinoma (A, B 200×).

A 66-year-old woman was admitted to hospital with unexplained intermittent dyspnea and hoarseness that had persisted for 1 month. The Karnofsky score was 90. The patient had no history of chronic diseases and family medical history. The left vocal cord and right vocal cord tumor resection were performed by endoscopic low-temperature plasma knife one and half a month ago. The postoperative histopathology showed the tumor cells were an invasive, highly differentiated SCC on the left vocal cord and squamous dysplasia on the right vocal cord, which conformed to the typical features of well-differentiated invasive squamous cell (Fig. 1). Distant metastasis was not found in the imaging assessment. According to the American Joint Commission on Cancer (AJCC) 8th edition staging system, the patient was diagnosed as TIbN0M0.

**Figure 2:**
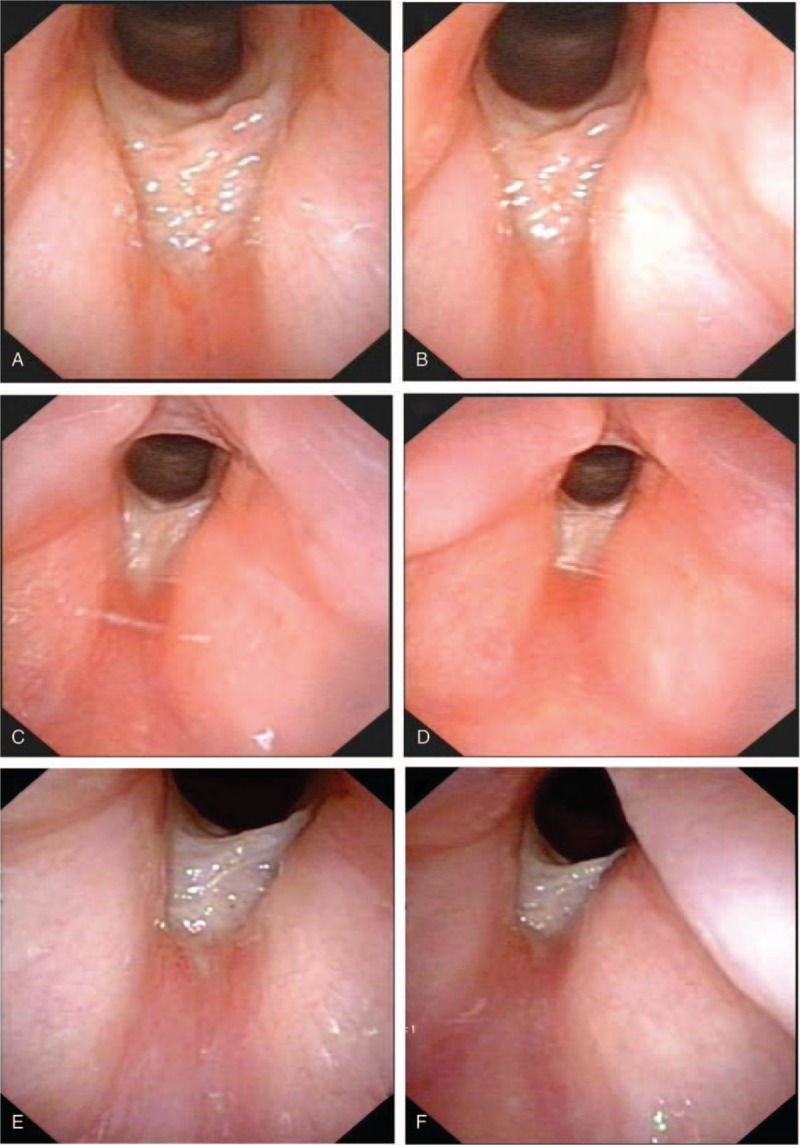
Laryngoscope. Granulation formation and scar formation in the glottis area before reoperation (A, B); after radiation DT20Gy/10f, the laryngoscope showed a white pseudo membranous was attached to the glottis(C, D); after radiation completed, no swelling was observed in the epiglottis, the white pseudo membrane can still be seen on the glottis (E, F).

**Figure 3:**
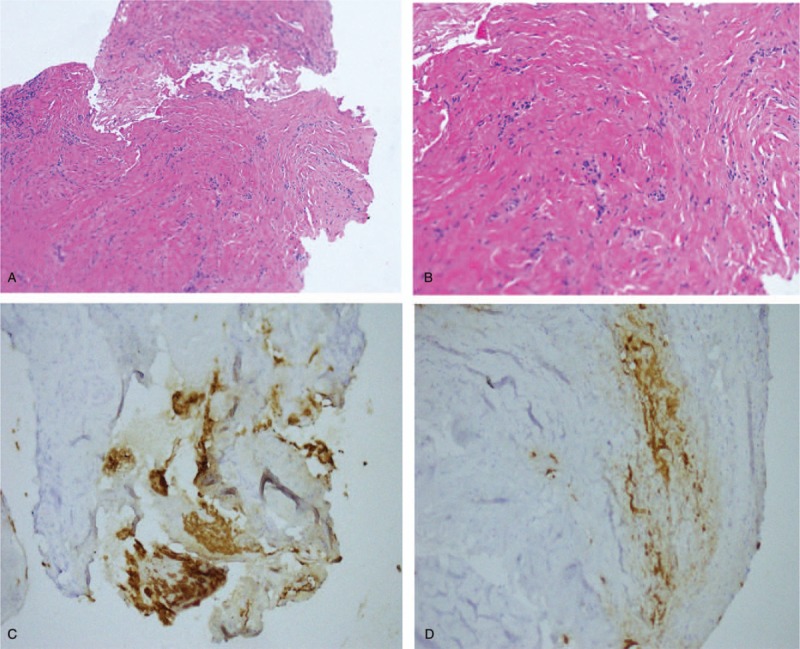
Hematoxylin and eosin staining (reoperation): hyperplastic fibrous tissue, focal epithelium atypical hyperplasia, in which chronic inflammatory cells can be seen (A: 100×; B: 200×); immunohistochemistry: immunohistochemical staining revealed positive staining for CK-pan (C, D: 200×).

After surgery of laryngeal cancer, the patient suffered intermittent dyspnea and hoarseness for nearly 1 month. Then the patient was examined by laryngoscope. Laryngoscope showed a mass on the glottis (Fig. 2A, B). The patient underwent pathological biopsy and resection of the mass with the guidance of laryngoscope under general anesthesia. Postoperative pathological examination showed (laryngeal) hyperplastic fibrous tissue, and chronic inflammatory cells can be seen in focal epithelium atypical hyperplasia. Immunohistochemistry also showed CK-pan [+], CgA [–], Syn [–]. Combined with immunohistochemistry and pathology, the patient was diagnosed with laryngeal granulation (Fig. 3). Intensity-modulated radiation therapy (IMRT) was performed for the patient within 24 hours after the surgery. The computerized treatment planning system (Eclipse) was utilized to determine radiation fields. The clinical target volume (CTV) was defined as the full throat; the planning target volume (PTV) included the CTV plus a 0.5 cm margin. The patient was treated with 6 MV X-ray using True Beam linear accelerator. The radiation dose was 2.0 Gy per fraction, with a total dose of 50 Gy in 5 fractions administered over a week. After 10 fractions, the patient suffered from odynophagia and a little coughing which were relieved after symptomatic treatment and the laryngoscope showed a white pseudo membranous was attached to the glottis (Fig. 2C, D). After RT, the throat of the patient experienced some discomfort and laryngoscope was applied and reexamined, indicating that the white pseudo membrane could still be seen on the glottis, which was considered as mucosal damage caused by RT. One month after RT, laryngoscope was routinely reexamined and the glottis area was smooth and there was only a little scar under the glottis, which did not affect sound function (Fig. 2E, F). It has been 3 years since the end of RT, and the patient's laryngeal granuloma has never recurred.

## 3 Discussions

In our PubMed search, there are some cases of primary vocal granuloma, albeit rare. But our case is the first reported case of glottic granuloma occurring after laryngeal neoplasm operation. When it comes to laryngeal granuloma, Clawsen first described laryngeal granulomas resulting from endotracheal intubation, and started to draw others’ attention to the study of laryngeal granuloma in 1932.^[8]^ Most of the times, granulomas are unilateral or bilateral tumor-like lesions with a smooth or irregular surface and a pedicle. The implantation pedicles are projected into the posterior area of the glottis, especially on the vocal apophysis.^[9]^ As we know, the reasons for the occurrence of larynx granuloma are controversial.^[1]^ Rintaro Shimazu studied experimental rats of gastroesophageal reflux disease (GERD) with mechanically injured vocal cord mucosa and demonstrated that the cause of laryngeal granuloma may be the combination of 2 factors: gastric reflux and mechanical injury.^[10]^ Martins RH clarified that Intubation granulomas occur more frequently after prolonged intubation, especially in females.^[9]^ Besides the aforesaid factors, voice abuse is also the frequent factor for laryngeal granuloma.^[2]^ In our case, the patient is a female and suffered prolong intubation during the radical surgery of laryngeal cancer, both of which may contribute to the formation of laryngeal granuloma.

Clinical treatments of granuloma, according to many academic literature, may consist of surgical excision, reflux treatment, voice therapy and the other approaches targeting predisposing factors.^[11,12]^ Surgery is still the predominant treatment for granuloma. However, patients with granuloma submitted to only sugery have high recurrence rates, ranging from 50% to 92%.^[10,13]^ Duan Hong-Gang studied that the repeated operation had no significant effect on recurred patients.^[14]^ The treatment of laryngeal granuloma had to be treated by surgery together with other treatments. Duan Hong-Gang found that combination surgery with proton pump inhibitors (PPIs) can reduce the recurrence from 50% to 38.4%.^[14]^ Yongli's survey had demonstrated the effectiveness of surgical removal followed by immediate radiation therapy in the management of refractory vocal process granuloma (VPG). In Yongli's study, low-dose RT combined with surgery resulted in a recurrence rate of 0% during the follow-up examinations of the next 3 to 6 years.^[1]^ With recent studies, postoperative RT with low-dose has showed great advantages in treating non-malignant disease.^[15,16]^ In our case, postoperative RT was necessary to be done.

The pathology of laryngeal granuloma is hyperplasic fibrous tissue, the formation of which is same as benign proliferation of normal tissues, such as skin keloid.^[1,17]^ During Several hours after the surgery, the granulation began to form. The main components of the granulation were the immature fibroblasts, the unstable collagen fibers and the new blood capillaries. All of them are sensitive to the radiation.^[1]^ The postoperative RT within 24 hours can effectively depress the reprolification of the lesion. Our patient was treated with RT immediately after surgery of laryngeal granuloma.

With rare reported accidents, there is no consensus in the literature regarding the best dose for laryngeal granuloma happening after laryngeal cancer. Usually, the low-dose RT (10–30Gy) is sufficient for unspecified laryngeal granuloma.^[1]^ For T1 glottic SCC, the optimal treatment strategy is not well-defined. In a single-institution retrospective analysis of 244 patients with T1–2 glottic SCC,^[18]^ the 5-year RFS was higher for patients who received RT as any part of their treatment (either definitive or adjuvant) than for those who underwent surgery alone (80% vs 65%, *P* < .01). Especially, in patients with T1 primaries, the 5-year RFS rates with primary RT vs. surgery were 83 and 75%, respectively (*P* = .05). In our case, we should both reduce the risk of recurrence rate of the laryngeal cancer and eliminate laryngeal granuloma concurrently. As for the first case of this kind, the dose of postoperative RT needed to be safely handled. After many discussions, the patient was given 50 Gy, namely, preventive dose, which cannot only decrease the recurrence of laryngeal granuloma and laryngeal cancer, but also prevent secondary malignant mucosa caused by high-dose RT.^[1]^ Now the symptoms including intermittent dyspnea and hoarseness have disappeared and the patient is currently alive with no evidence of recurrence.

## Author contributions

**Conceptualization:** Jingyi Wu.

**Funding acquisition:** Lijuan Ding.

**Investigation:** Jingyi Wu, Lijuan Ding.

**Methodology:** Lijuan Ding.

**Project administration:** Lijuan Ding.

**Resources:** Lijuan Ding, Lihua Dong.

**Software:** Yu Wu, Lijuan Ding, Lihua Dong.

**Supervision:** Lijuan Ding.

**Validation:** Lihua Dong.

**Visualization:** Lihua Dong.

**Writing – original draft:** Jingyi Wu.

**Writing – review & editing:** Jingyi Wu, Tongchao Jiang.
